# Health literacy skills for informed decision making in colorectal cancer screening: Perceptions of screening invitees and experts

**DOI:** 10.1111/hex.12658

**Published:** 2017-12-20

**Authors:** Anke J. Woudstra, Daniëlle R. M. Timmermans, Ellen Uiters, Evelien Dekker, Ellen M. A. Smets, Mirjam P. Fransen

**Affiliations:** ^1^ Department of Public Health Academic Medical Center Amsterdam Public Health research institute University of Amsterdam Amsterdam The Netherlands; ^2^ Department of Public and Occupational Health and the EMGO institute for Health and Care Research VU University Medical Center Amsterdam Public Health research institute Amsterdam The Netherlands; ^3^ National Institute for Public Health and the Environment (RIVM) Bilthoven The Netherlands; ^4^ Department of Gastroenterology and Hepatology Academic Medical Centre University of Amsterdam Amsterdam The Netherlands; ^5^ Department of Medical Psychology Academic Medical Center Amsterdam Public Health research institute University of Amsterdam Amsterdam The Netherlands

**Keywords:** colorectal cancer screening, conceptual framework, context‐based measurement, health literacy, Informed decision making

## Abstract

**Background:**

The process of informed decision making (IDM) requires an adequate level of health literacy. To ensure that all individuals have equal opportunity to make an informed decision in colorectal cancer (CRC) screening, it is essential to gain more insight into which health literacy skills are needed for IDM. Our aims were (i) to explore how individuals make a decision about CRC screening and (ii) to explore which skills are needed for IDM in CRC screening and (iii) to integrate these findings within a conceptual framework.

**Methods:**

We conducted 3 focus groups with individuals eligible for CRC screening (n = 22) and 2 focus groups with experts in the field of health literacy, oncology and decision making, including scientific researchers and health‐care professionals (n = 17). We used framework analysis to analyse our data.

**Results:**

We identified and specified ten health literacy skills, which varied from the ability to read and understand CRC screening information to the ability to weigh up pros and cons of screening for personal relevance. The skills were linked to 8 decision‐making stages in CRC screening within a conceptual framework. We found differences in perceptions between screening invitees and experts, especially in the perceived importance of CRC screening information for IDM.

**Conclusions:**

This study provides insight into the decision‐making stages and health literacy skills that are essential for IDM in CRC screening. The proposed conceptual framework can be used to inform the development of context‐based measurement of health literacy and interventions to support IDM in cancer screening.

## 1 INTRODUCTION

Colorectal cancer (CRC) accounts for one of the most common causes of cancer deaths worldwide. Early detection of precursors as well as pre‐clinical stages of CRC can reduce morbidity and mortality of the disease in the population.^1^ It is increasingly being recognized that screening programmes should facilitate informed decision making (IDM) about participation rather than pursuing increased uptake, as screening involves potential harms and benefits on an individual level.^2^

Informed decision making has been described by Timmermans as a process that includes 4 decision‐making stages: (i) becoming aware of the decision to be made, (ii) structuring decision options and outcomes, (iii) evaluating decision options for personal relevance and (iv) making the final decision.^3^ An informed decision is considered to be the outcome of these stages and is commonly defined as one that is “based on relevant knowledge, consistent with the decision‐maker's values and behaviourally implemented.”^4^, ^5^ Accordingly, screening invitees are explicitly required to deliberate harms and benefits to make an informed decision about participation in CRC screening. As IDM relies on the application of information to one's own situation, it is assumed that this decision‐making process requires an adequate level of health literacy.^6^

Health literacy has been described by Sørensen et al^7^ as 4 aspects of information processing (access, understand, appraise and apply). *Access* refers to the ability to seek, find and obtain health information. *Understand* refers to the ability to comprehend the health information that has been accessed. *Appraise* refers to the ability to interpret, filter, judge and evaluate the health information that has been accessed. *Apply* refers to the ability to communicate and use the information to make a decision to maintain and improve health. Health literacy is dependent on health‐care content and context. For instance, the decision‐making process for CRC screening, in which individuals need to understand the disease characteristics of CRC and weigh up the pros and cons of screening, requires different health literacy skills than diabetes self‐management, in which individuals have to adhere to medication regimes and maintain a healthy lifestyle.^8^ The conceptualization of health literacy skills has implications for its measurement and consequently, for intervention development.^9^

Decision making about CRC screening is complex and it remains an ongoing challenge to inform all screening invitees in an accessible and understandable way.^10^ To ensure that all individuals have equal opportunity to make an informed decision in CRC screening, it is important to understand which health literacy skills are perceived as essential for IDM in this particular context. This knowledge is necessary for the development of context‐specific health literacy measures and tailored interventions that support IDM in screening. While informed decisions seem to be a central outcome in the conceptualization of health literacy, few studies have explored the integration of models of IDM and health literacy.^6^, ^7^ In addition, there is a paucity of research on what exactly constitutes an informed decision in cancer screening from the perspective of screening invitees. The aims of this study were therefore (i) to explore how individuals eligible for screening make a decision about screening and (ii) to gain more insight into which health literacy skills are needed for IDM about CRC screening and (iii) to integrate these findings within a conceptual framework.

## 2 METHODS

### 2.1 Study design

We conducted 2 focus groups with experts representing different fields of expertise (health literacy, oncology and decision making) including scientific researchers and health‐care professionals (n = 17) and 3 focus groups with individuals eligible for CRC screening (n = 22). The focus group interviews were moderated by MF from May to October 2015 in the Netherlands, where a nationwide CRC screening programme is being implemented since 2014 (see Box 1). An advantage of focus groups is their use of group dynamics to stimulate the discussion,^11^ which provided us with deeper insights into individuals’ considerations about CRC screening participation. According to Dutch law, this study was waived from requiring medical ethical approval. Nonetheless, we guaranteed the anonymity of the participants and ensured that informed consent was obtained from all participants prior to conducting the focus group interviews.

Box 1 **Background information on Dutch CRC screening programme**

1 In the Netherlands, a nationwide colorectal cancer (CRC) screening programme has been implemented since 2014. By 2019, all individuals between the ages 55‐75 years will be invited every 2 years to perform an immunochemical faecal immunochemical test (FIT) at home. The CRC screening invitation includes an announcement letter (which is sent 2 weeks prior to the invitation), a leaflet about CRC and CRC screening, a leaflet including instructions about performing the FIT, the FIT and an answer form. Additional information about CRC screening is available on the website of the National Institute for Public Health and the Environment.^1^ When the FIT is positive (ie blood is found in the stool sample), individuals are invited for further diagnostic follow‐up: a colonoscopy.

### 2.2 Focus group interviews with experts

The experts (scientific researchers and other professionals) were recruited from the working group “Shared Decision Making” at the Academic Medical Centre in Amsterdam, the Netherlands (n = 8), and the working group “Scientific research” of the Dutch Health Literacy Alliance (n = 9; Table 1). The Dutch Health Literacy Alliance was established in 2010 to (i) raise awareness about health literacy among policy makers, health professionals, health‐care institutions and the general public, (ii) facilitate exchange of knowledge and expertise about health literacy research and (iii) integrate health literacy issues in education, research and patient participation.^12^

**Table 1:** Characteristics of focus groups with experts and individuals eligible for CRC screening

**Experts (N=17)**	
Scientific researchers N (%)	11 (65)
Oncologists N (%)	4 (24)
Computer scientist N (%)	1 (6)
Communication consultant N (%)	1 (6)
**Target group (n=22)**	
Female N (%)	8 (40)
Age (mean, min‐max)	68 (61‐73)
Educational level N (%)	
High (University/College)	10 (45)
Middle (Intermediate vocation/Higher general secondary education/Pre‐university education)	4 (18)
Low (Primary school/Lower general secondary education)	8 (36)
Low HL (NVS < 4) N (%)^a^	7 (30)
Non‐Dutch ethnic background N (%)	9 (40)
Participated in CRC screening N (%)	4 (18)

a NVS scores missing (N=4). CRC, colorectal cancer; NVS, newest vital sign.

Starting from the decision‐making model of Timmermans^3^ and the conceptual model of health literacy of Sørensen et al,^7^ which are both well‐established models, we developed the topic guide for the focus groups (Box 2). At the beginning of the focus groups, MF presented Timmermans’ model to the experts. The specific aims of the interviews with the experts were to examine (i) whether and how the decision‐making stages are used in practice, (ii) which decision‐making stages and health literacy skills are minimally required for IDM and (iii) whether other stages of decision making should be considered for IDM about CRC screening participation.

Box 2 **Topic guide focus groups with experts—key discussion questions**

1 Decision‐making stages:

1. Which decision‐making stages come to mind when thinking about decision making in CRC screening?
2. How do these decision‐making stages (awareness, perception, evaluate, make a decision) reflect decision making about CRC screening in practice?
3. What decision‐making stages do you think should be added or removed?

Health literacy skills:

1. Which skills do you think are required to move from 1 stage to another?
2. Which skills do you think are minimally required for informed decision making in the context of CRC screening?

### 2.3 Focus group interviews with the target group

Five hundred individuals (aged 55‐75 years) were recruited from general practices in the southeast of Amsterdam. They were randomly selected and invited by postal mail. One hundred and forty of them expressed interest to participate in research on CRC screening (by means of a prepaid response card). Of these 140 individuals, 46 were contacted for another study on knowledge of CRC screening.^10^ Of the remaining 94 individuals, we were able to contact 41 of them by phone. We were unable to reach the other 53 individuals in the previous study or current study. Twenty‐two individuals finally participated in the 3 focus group interviews (Table 1). The total number of included participants was based on data saturation. Two focus groups were conducted at the Academic Medical Centre in Amsterdam, and 1 focus group was conducted in a general practice in the southeast of Amsterdam.

The specific aims of the focus groups were as follows: (i) to examine which decision‐making stages and health literacy skills are being used in decision making about CRC screening and (ii) to examine which stages and skills were perceived as being essential to make an informed decision about CRC screening participation. During the focus group interviews, participants were given the opportunity to ask questions about the CRC screening information. To gain insight into participants’ basic health literacy skills, the Dutch version of the Newest Vital Sign (NVS‐D)^13^ was administered at the end of each focus group.

### 2.4 Data analysis

All focus groups were audio‐recorded and transcribed. The interviews were analysed using framework analysis by the authors AW and MF.^14^, ^15^ In a framework analysis, the first step is to familiarize with the data (*familiarization)*. MF conducted the focus group interviews and AW transcribed and re‐read the transcripts. For the second step (*thematic analysis),* we developed a coding scheme using decision‐making stages and health literacy skills as themes using qualitative data analysis software (MAXQDA version 12, VERBI GmbH, Germany). We applied these codes to the focus group transcripts (*indexing)* and categorized these codes to summarize our findings (*charting)*. This was essentially a comparative process, by which the different transcripts from the individuals eligible for CRC screening and the experts were compared with each other and analysed to find relationships between codes. Agreement on the codes was reached between AW and MF during weekly meetings.

## 3 RESULTS

### 3.1 Characteristics of study population

Of the seventeen experts, all but 1 was born in the Netherlands. According to the Newest Vital Sign (NVS), 7 of the twenty‐two individuals eligible for screening had lower health literacy (NVS score < 4). Four participants were unable to complete the NVS; 1 was illiterate, 1 reported to have forgotten her reading glasses and 2 participants had to leave before the end of the focus group and were therefore unable to fill out the form.

### 3.2 Findings from the focus groups in conceptual framework

**Figure 1:**
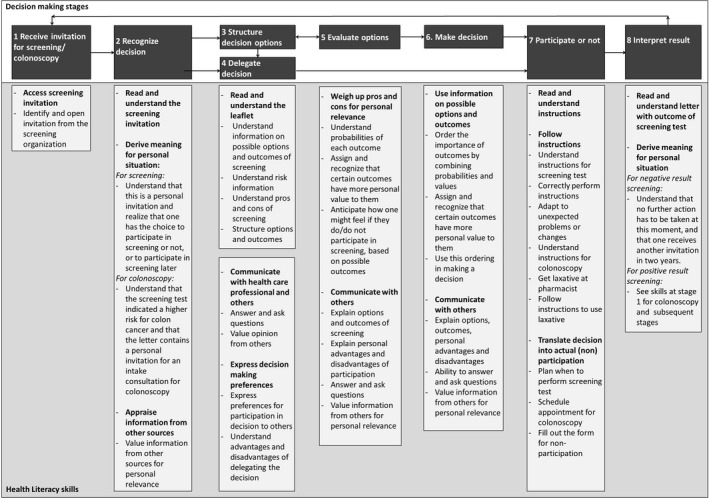
Conceptual framework of decision‐making stages and health literacy skills in colorectal cancer screening

We used the model by Timmermans^3^ and the model by Sørensen^7^ as a guide for the analysis of the findings from the focus groups. By integrating our focus group findings within these models, we developed a novel conceptual framework that combines decision‐making stages and health literacy skills in the context of CRC screening (Figure 1). We identified 8 decision‐making stages and specified 10 key health literacy skills that were deemed essential for IDM in CRC screening. These decision‐making stages (marked in bold) and health literacy skills (marked in italics) are described in detail below. The selection of quotes that are representative of the decision‐making stages and health literacy skills is described in Tables 2 and 3.

**Table 2:** Example quotes—Perceptions of experts

Stage 1. Receive invitation screening/colonoscopy	Quote 1: “You do need the skill to make a decision about that [whether or not to participate]. If you don't recognize the envelope as being a choice and it ends up in the bin, then you've missed it because you don't have the skills.” (Shared Decision Making focus group)
Stage 2. Recognize decision	Quote 2: “First it should be clear that people can say ‘I'll do it or I won't do it’. That has to be clear first, because many people think it's obligatory…that you have to obey the call. No, there is a choice—and many things are dependent on that. Thus, first, you have the choice, then, so what is this choice—what if I participate, what if I do not participate—the pros and cons.” (Shared Decision Making focus group)
Stage 3. Structure decision options and outcomes	Quote 3: “That [structuring of options and outcomes] is tricky because people have probably heard about it via the news‐ that the invitation is coming—and they have already made a decision… Would you then still let yourself become informed?” (Shared Decision Making focus group)
	Quote 4: “I miss the [knowledge] check as to whether the information has been properly understood. For many things we've seen recently concerning the provision of information—there's a brief test in which the patient answers some questions which clarifies whether or not they've understood the information. That's what I miss here—I think that is essential.” (Shared Decision Making focus group).
	Quote 5: “You would like to know how people will participate on the basis of what. So maybe there should a test about the pros and cons… [This would enable an invitee to] go back, to get more information. So, that they make a decision based on [the right CRC screening] information.” (Scientific research group)
	Quote 6: “In the leaflet, there should be a page with a test to check whether you have understood it, like the basics.” (Shared decision making focus group)
Stage 4. Delegate decision	Quote 7: “People have many things that they need to make a decision about—especially if you have comorbidity. I can imagine that it's rather difficult. So, just go to the GP, discuss with him and do shared decision making, or tell him ‘I want you to make a decision.’ For this group, that's sufficient—I think it's also a good informed decision if you realize that [based on all this] I want to go to my GP.” (Shared Decision Making focus group).
	Quote 8: “[In order to delegate their choice] they first need to understand that they have a choice.” (Scientific research focus group)
Stage 5. Evaluate options based on facts and/or feelings	Quote 11: “What if the decision can't be made because of lack of skills? That people think: oh, faeces, a test, I cannot do this, I won't bother […] Actual participation … I can imagine that invitees will be held back [due to barriers], or people will be deterred from actually participating in colorectal cancer screening, while in fact they would actually like to participate.” (Scientific research focus group)
Stage 6. Make decision	Quote 9: “You can inform them so that they apply those skills to make a decision—but they can also put the information aside when, for example, their neighbour has cancer. In this case, they are informed but they do not use the information. “(Shared Decision Making focus group)
	Quote 10: “Basing decisions on feelings or a habit, tradition, what's basically wrong with that?” (Scientific research focus group)
Stage 7. Participate or not	Quote 12: “At a certain moment, you'll get that people do not want to participate if they are being forced [to weigh up all the pros and cons].” (Scientific research focus group)
Stage 8. Interpret result	Quote 13: “The choice for a colonoscopy [should be] included [in the conceptual framework] and I think that's only right and logical since this is a consequence [of the first part—the FIT]” (Scientific research group)

**Table 3:** Example quotes—Perceptions of individuals eligible for screening

Stage 1. Receive invitation screening/colonoscopy	Quote 1: Interviewer Would you open this envelope? Participant Of course—why not? This [colorectal cancer screening] interests me. Even without this information I would participate. I think that's true for most people. Interviewer If you read this [invitation] letter, what would you think? Participant Well, I think I should immediately participate. (Male, age = 67, NVS=3)
	Quote 2: “There are certain neighbourhoods where such an invitation simply disappears between the old newspapers—they don't even open it. But these aren't the people who sit at this table right now.” (Male, age 66, NVS = 6)
Stage 2. Recognize decision	Quote 3: Interviewer How would you make a decision? Participant I don't understand that question because I don't think there are cons of screening. You have faeces every day, so what does it matter? (Female, age 72, NVS = 4)
	Quote 4: Interviewer Would you like to search for more information? Participant I always do that for my medical things. Then I google the word, I put it in Google and then see what comes up. Then I see whether it's a website that I want to read, because there's no use in just hearing a load of drama. So, if it looks like a reasonable website or whatever, then I'll see what they say about it […] Then you have to stop—because if you don't watch out, you get flooded with information. Interviewer Why would you search for additional information? Participant Because very few things are really complete. I mean, you need to search a bit for yourself. That's not intended as a recommendation to put more information in it [in the leaflet] —that is not what I mean. But I just want to have the feeling that I've found something for myself—that I've formed a picture myself. (Female, age 71, NVS = 6)
	Quote 5: Participant I think I would select information from the leaflet. Interviewer Why would you select information? Participant Laziness—I mean, I don't need any redundant information. (Female, age 72, NVS =4) .
Stage 3. Structure decision options and outcomes	Quote 6: Participant 1 Why would you postpone this? (Female, age 73, NVS=2) Participant 2 This is something you don't postpone. (Male, age 69, NVS=5)
	Quote 7: “It's actually minimal [not much trouble to perform the FIT] compared to the trouble it can cause [having CRC].” (Female, age 69, NVS=6)
	Quote 8: “Perhaps my decision is influenced by my wife—but actually that doesn't really influence me because I have already made the decision.” (Male, age 73, NVS=6)
Stage 4. Delegate decision	Quote 9: “I might have done that [making the decision] with my previous GP but I don't know this one at all and she doesn't know me, so I would make the decision myself.” (Male, age 73, NVS=6)
	Quote 10: “It says here that a tube will be sent to your home and that you have to use this to put some faeces in. In other words, you do it at home and it's not a big deal.” (Male, age 73, NVS=6)
	Quote 11: “I am always in favour of screening programmes—if it's not too serious. I would absolutely take the first step [the FIT], but I am not sure about the second step. That sort of depends…I would participate, but I would also just wait for the intake.” (Male, age unknown, NVS=4)
	Quote 12: “If you give a lot of information [about the cons], then people tend to get ahead of themselves—and it's so important that people do participate.” (Female, age 69, NVS=6)
	Quote 13: “I think you'd better leave it [the cons of screening] out of the leaflet, because it scares me. I don't really know—but I need to think again about whether I want to participate.” (Female, age 64, NVS= 0)
Stage 5. Evaluate options based on facts and/or feelings	Quote 14: “I would say that my feelings don't really play a role in this—because every time there's a conversation about deadly diseases, I want to keep away from it. My feelings say: push it away—but my mind says: this is very important because I want something to be done if it's really necessary. So when we talk about feelings, I prefer to leave my feelings out of it.” (Female, age 67, NVS=6)
	Quote 15: I've known people at work, and also people that are close to me—I've seen them die and [I've seen] those that survived colorectal cancer. So, I know how important it is. It's not a difficult decision [to participate]. (Female, age 62, NVS=5)
	Quote 16: Participant When I read this letter [information leaflet], then I see about 4‐5 per cent of the people in the Netherlands have colorectal cancer. That's really scary. Interviewer What do you think when you read this? Participant Well, I think I should participate straight away. (Male, age 67, NVS=3)
Stage 6. Make decision	Quote 17: “I would read the leaflet, but I always show it to my sister and ask her—what do you think?—and sometimes I overlook something and then she tells me that I didn't see this and that, and how I really need to do it.” (Female, age 64, NVS=0)
Stage 7. Participate or not	Quote 18: Interviewer When you read the invitation, what would you do? Participant I would participate. Of course I would participate. Interviewer But you won't know what the screening is about. Participant But you can decide later, right? Because here it's just whether you want to send the test [send the FIT to the laboratory] and then you can make a decision. (Female, age 62, NVS=5)
Stage 8. Interpret result	Quote 19: “I didn't know that there were disadvantages of participating in screening. I just read this. I would think about it more… Not to do the test [the FIT] but to do the follow‐up [colonoscopy]. They give you a colonoscopy and then there could be complications. I did not think about that. You should certainly participate, but I would think about it more.” (Female, age 64, NVS=0)
	Quote 20: “The disadvantages only appear when you participate in the follow‐up. You can always decide later.” (Male, age 66, NVS=6).
	Quote 21: “If you participate and there is blood [FIT test is positive], then it's more difficult to make a decision and say: I won't participate. So I think that if you don't participate at all, that makes things easier.” (Female, age 71, NVS=6)

#### 3.2.1 Perceptions of experts Process of decision making and health literacy skills

In general, experts (including scientific researchers and other professionals) stated that the process of decision making about CRC screening starts with **receiving the screening information (stage 1)**. Yet, this was also perceived as being insufficient for making an informed decision. Although the invitation is sent to the invitees’ homes via postal mail, experts mentioned the importance of having the skills to actually *access the information* by opening, reading and understanding the letter. For accessing the screening invitation, experts mentioned that invitees *need to identify the screening invitation as being a choice* (Quote 1, Table 2). Even though the standard information in the Dutch CRC screening programme was perceived as being sufficient for making an informed decision, experts stated that invitees might search for additional information on CRC screening, for which they would also need the skills *to value and judge information*. Hence, information about CRC screening, other than the standard information, might also facilitate IDM about participation according to the experts.

With regard to **stage 2 (Recognizing that there is a decision to be made)**, experts pointed out that invitees should at least *be aware of CRC screening and understand the information*, also to allow making an informed decision about non‐participation (Quote 2, Table 2). The decision options were agreed to be participation, non‐participation or postponing participation.

**Structuring these decision options (stage 3)** was believed to be an essential stage for *understanding that the decision to be made is voluntary*. In addition to this, experts mentioned that screening invitees need to be *able to ask and answer questions* about the screening information if they do not understand the information. These were considered to be 2 different abilities. The majority of the experts perceived **structuring the decision options (stage 3)** to be an important stage in achieving IDM, yet this was also believed to be the most difficult one, especially when invitees are already aware of CRC screening (Quote 3, Table 2).

In addition, experts from both focus groups mentioned the importance of adding a “knowledge check” for **structuring the decision options and outcomes (stage 3).** Accordingly, one expert stated that when invitees are about to make a decision, it needs to be checked whether they have really understood the information. This would also help invitees to make a decision based on the “right” CRC screening information (Quote 4‐5, Table 2). Nevertheless, there was some confusion among the experts about who should perform the knowledge “check” as there is no direct involvement of a health‐care provider in the national CRC screening programme. An expert from the shared decision‐making focus group eventually stated that there should be a “check” in the CRC screening leaflet (Quote 6, Table 2).

Most experts also mentioned that **delegation of the decision (stage 4)** to a health‐care professional may lead to IDM, provided that screening invitees are able to structure the potential harms and benefits of CRC screening for personal relevance (Quote 7, Table 2). Furthermore, when screening invitees prefer to **delegate their decision (stage 4)**, they should still be able to *understand that there is a decision to be made about participation* (Quote 8, Table 2). In all decision‐making stages, experts mentioned that screening invitees should also be *able to ask for help* with reading or translation, especially if they have difficulty understanding the information materials themselves. The involvement of peers, siblings, family and other non‐professionals in the decision‐making process would then require the ability to *value and judge information from others for personal relevance*.

Likewise, experts mentioned that invitees might **make the decision (stage 6)** based on feelings or experiences (Quote 9, Table 2). This was perceived as being inevitable and not necessarily as something that would lead to a less informed decision (Quote 10, Table 2). Several experts mentioned that invitees should derive meaning from the screening information for their own situation, which characterizes **stage 5 (Evaluation of these decision options).** Hence, in addition to the *ability to understand the information*, experts mentioned the importance of *understanding risk information and deriving meaning for one's personal situation*. Moreover, a number of experts mentioned that even though some screening invitees might have the intention to participate, they may not be able to actually participate (Quote 11, Table 2). The decision‐making stage **actual participation (stage 7)** was therefore believed to require not only *the ability to understand the instructions* but also overcoming possible perceived barriers to screening. Experts mentioned that if invitees do have the intention to participate, but are not able to, participation may become a matter of accessibility to screening, rather than it being an individual's decision. One expert from the scientific research focus group believed that rational IDM—in which individuals need to weigh up the potential harms and benefits of screening—might deter people from making an informed decision about CRC screening participation (Quote 12, Table 2).

Several experts emphasized that *the ability to understand instructions in the leaflet* and *ask for help if necessary* is essential in making an informed decision. In addition, screening invitees would need to be able to *anticipate regret* when making a decision. For example, the decision not to participate also requires particular health literacy skills according to several experts. That is, screening invitees need to be *able to fill out a form and anticipate the consequences of non‐participation*. Experts in the scientific research focus group also reported that there is a difference between skills that are needed for *making a decision* and skills that are needed for *following instructions*. Different skills are also required for *following the instructions for the FIT* and for *making an appointment for a colonoscopy*. Moreover, they mentioned that it is important that screening invitees understand the whole screening procedure, including the FIT and the follow‐up diagnostic test (Quote 13, Table 2). This was deemed to be important for the stage **interpretation of the result (stage 8).**

#### 3.2.2 Perceptions of individuals eligible for screening: Process of decision making and health literacy skills

There was a wide variety in the level of awareness about the national CRC screening programme among the individuals eligible for screening. While the majority of the participants had heard about CRC screening prior to the interview and 4 of them had already participated, for a number of them CRC screening was completely new. With regard to the **first stage (Receiving invitation)**, all participants mentioned that they would certainly open the screening invitation (Quote 1, Table 3), yet they could imagine that individuals in their surroundings would discard the letter because they would not recognize this as being important. One participant also pointed out the challenge of uncovering perceptions of individuals that are unlikely to open the screening invitation (Quote 2, Table 3).

For the majority of the participants, participation was self‐evident. Hence, they did not always **recognize that there is a decision to be made (stage 2;** Quote 3, Table 3). All participants understood that participation was voluntary, yet they did not always understand why people would not want to participate. While the experts emphasized the importance of having the ability to understand the whole screening procedure and to interpret the results, the majority of the individuals eligible for screening thought of screening as a procedure that consists of 2 steps. They would first make a decision about performing the FIT and then they would decide upon undergoing a colonoscopy, but only after receiving a positive FIT result. For **stage 2 (Recognize decision),** there was a difference in preferences for seeking additional information to understand what the decision is about. While some participants preferred to postpone the decision until they would receive the invitation, some had already searched for CRC screening information online. One participant's search for information could be viewed as a sign of autonomy (Quote 4, Table 3). A number of participants reported that they would purposely select information from the leaflet, suggesting that the length of the leaflet might be too burdensome (Quote 5, Table 3).

While the experts came up with the following **decision options to be structured (stage 3):** participate, not participate or postpone participation, the latter was not always recognized as being an option among individuals eligible for CRC screening (Quote 6, Table 3). The vast majority of the participants were positive towards CRC screening (Quote 7, Table 3). Statements concerning **evaluating and structuring options and outcomes (stage 3 and 5)** often involved consideration of others (Quote 8, Table 3). Some participants mentioned that the decision to participate follows from a moral responsibility towards their family members. Accordingly, a number of participants mentioned that they would participate immediately, without deliberately weighing up the pros and cons of screening.

Although most participants did not mind talking with health‐care professionals and informal others about CRC screening, they stated that they would prefer to make the decision about participation on their own. **Delegation of the decision (stage 4)** to a professional was often dependent on the relationship they had with their health‐care provider (Quote 9, Table 3).

While experts mentioned the importance of having the ability to *understand and balance risk information* for **stage 5 (Evaluating the decision options),** almost all of the individuals eligible for CRC screening believed that there were no risks involved when performing the FIT (Quote 10, Table 3). By contrast, the follow‐up procedure (colonoscopy) was believed to be potentially harmful (Quote 11, Table 3). For some participants, the risk information in the CRC screening leaflet felt too free of engagement, stating that they would like more guidance in what the risks would mean for them personally. Some participants preferred more straightforward information about participation or not, indicating that the information about the pros and cons was too difficult for personal deliberation. In addition, some participants were rather reserved about the provision of further information about the potential harms of CRC screening, as this was believed to discourage participation (Quote 12‐13, Table 3). For **stage 5 (Evaluating the decision options)**, a number of participants reported that they preferred to base their decision on factual information rather than on feelings. Decisions that are based on feelings, rather than on facts, were often believed to result in uninformed decision making. This was exemplified by participants’ statements on how the involvement of feelings in a decision‐making process may cause distress (Quote 14, Table 3). Nonetheless, participants’ accounts showed that personal values and feelings were naturally present in the decision‐making process (Quote 15, Table 3). Participants’ decision making was also influenced by their perceptions of CRC risk. For instance, some participants were rather alarmed by the reported risks of CRC in the screening leaflet and mentioned that this would be an important reason for them to participate (Quote 16, Table 3).

All participants mentioned that they understood the instructions for performing the FIT. Even without understanding all the decision options, the participants were confident that they would be able to **make a decision themselves (stage 6)** and perform the instructions for the FIT. For example, 1 respondent with low health literacy reported that she would ask help from her sister for following the instructions (Quote 17, Table 3). The barriers that were reported in participation were mostly of practical nature, such as costs, collecting faeces and sending the FIT to the laboratory. Nevertheless, those barriers were not considered to be very detrimental in making an informed decision. All participants were confident that they were able to **actually participate (stage 7;** Quote 18, Table 3).

Regarding **stage 8 (Interpretation of the result)**, the perceived harms were mostly related to the risks of having complications due to a colonoscopy but not to the risk of receiving a false‐positive or false‐negative test outcome. Hence, for a number of participants, the potential harms of CRC screening were not always evident (Quote 19, Table 3). This finding also pertains to stage 3 (where the understanding of the information begins) and stage 5 (using this information for the evaluation of the decision options). The idea that CRC screening may cause harm often came as a surprise (Quote 20, Table 3). Only 1 participant explicitly considered the consequences of the test outcomes for undergoing a colonoscopy (Quote 21, Table 3).

## 4 DISCUSSION

We identified and specified 8 decision‐making stages and 10 main health literacy skills for IDM in CRC screening and incorporated these within a conceptual framework (Figure 1). The health literacy skills represent abilities in accessing, understanding, deriving meaning, appraising information, communicating, weighing up pros and cons, using information, following instructions and translating decisions into actual participation. Based on our findings, we linked these health literacy skills to the following 8 decision‐making stages: (i) Receiving invitation, (ii) Recognizing decision, (iii) Structuring decision options, (iv) Delegating decision, (v) Evaluating options based on facts and/or feelings, (vi) Making a decision, (vii) Participating or not and (viii) Interpreting result. The framework reflects the importance of IDM as a broad social construct in the context of CRC screening, which includes multiple stages of decision making and different health literacy skills per stage. This study is the first to qualitatively explore the perceptions of experts and individuals eligible for CRC screening on health literacy skills and decision making, and has implications for further research and practice.

Our study revealed important differences in perceptions between individuals eligible for CRC screening and experts on the essential skills and decision‐making stages. First, our study showed how experts viewed IDM as the result of having the ability to access, understand, appraise and apply the CRC screening information for personal relevance. In other words, the experts viewed IDM as the result of having a broad range of health literacy skills. More specifically, experts reported that individuals should be able to deliberately weigh up the pros and cons of screening and balance risk perceptions about CRC and CRC screening. This seemed to be of lesser importance from the perspective of individuals eligible for screening, as most of them held participation to be self‐evident. For instance, the ability to structure and evaluate the decision options was less evident in the participants’ accounts. Individuals eligible for screening even expressed concerns about reporting the potential harms in the leaflet, as this could deter potential screening invitees.

We also found differences in perceptions about information needs between the experts and the individuals eligible for screening. While the experts mentioned that the standard screening information should be sufficient for making an informed decision, a number of individuals eligible for screening expressed preferences to search for additional information themselves. In line with these findings, a recent review underlined that actively seeking for health information may increase an individual's empowerment. However, the authors also mentioned the importance of guiding health information seeking as readily available online materials are often too complex for the general public to understand or to act on.^16^

Individuals eligible for screening reported the following barriers towards screening: costs, collecting faeces and sending the FIT to the laboratory. An important facilitator towards screening participation was the feeling of having a moral responsibility towards others. This finding of having a moral responsibility has also been shown in previous research in the context of prenatal screening.^17^ Although none of the screening invitees would want to delegate their decision about participation in CRC screening and perceived participation to be a personal decision, decision making often did involve the feeling a having a moral responsibility towards others.

While the experts emphasized the importance of understanding the whole screening procedure, many individuals eligible for screening would not linger over the consequences of participation in the first part of screening (ie performing the FIT). In fact, the screening procedure was often perceived as consisting of 2 separate decision‐making processes, in which the first (deciding about performing the FIT) was perceived as being free of risks and the second (deciding about undergoing a colonoscopy) was perceived as being potentially harmful. This perception of a 2‐step screening procedure might serve as 1 explanation for the difference in uptake between FIT and colonoscopy in the Dutch CRC screening programme. In 2016, more than 70% of the screening invitees participated in the CRC screening programme. However, about 20% of the participants who received a positive FIT test did not participate in the follow‐up procedure (a colonoscopy).^18^ Further research is needed to investigate how individuals’ perceptions of the screening procedure influence their decision about CRC screening participation.

Our study shows that common operationalizations of IDM^4^ do not necessarily meet the needs of all individuals eligible for screening. The assumption that minimum requirements for CRC screening information should be expert‐defined and similar to all, and result in deliberative decision making,^2^ may well prove counterproductive for certain groups, such as for those with lower health literacy. For instance, the weighing up of individual pros and cons may be perceived as being too complex. In addition, an explicit discussion of the potential harms and benefits may deter people from making an informed decision about screening participation. Further research should continue to unravel this area of tension for ensuring equal opportunity to make an informed decision in cancer screening for all individuals.

With regard to health literacy skills, our findings show that IDM in CRC screening does not only require functional health literacy skills (ie ability to read and write), which is hitherto assessed by the majority of health literacy measures.^19^ Although the ability to understand information was mentioned as being essential in all decision‐making stages, the experts perceived more advanced health literacy skills such as critical skills (ie ability to appraise) and interactive skills (ie ability to communicate with others)^22^ as being of equal importance. Additionally, experts emphasized the importance of the ability to ask for help for progressing through the decision‐making stages, especially for those who have difficulty with understanding screening information.

Our findings support previous research on the conceptualization of health literacy in health‐care contexts.^9^, ^20^ Smith et al, for example, showed that all 3 levels of health literacy as described by Nutbeam et al^8^ are equally important in decision making about CRC screening: functional health literacy skills are required for acquiring knowledge on screening, interactive health literacy skills are required for extracting information, communicating with others and expressing preferences for involvement and critical health literacy skills are required for critically thinking about the role and credibility of the information.^9^ Furthermore, Edwards et al's^20^ health literacy pathway model showed that health literacy is a multidimensional construct that develops over time, across different health contexts and through social interactions. By identifying the different stages and health literacy skills in CRC screening, our conceptual framework can be used to inform the development of health literacy measures in the context of cancer screening.

Although our findings did not reveal explicit differences between participants with low and adequate health literacy (as measured by the NVS‐D), previous research showed that IDM in CRC screening is a challenge for individuals with low health literacy as well as those with adequate health literacy.^21^ This finding underlines the importance of gaining more insight into which health literacy skills prove challenging for IDM, so that information in different decision‐making stages can be tailored to different health literacy levels. Similarly, screening organizations should consider using information strategies that are accessible for the varying health literacy levels of all screening invitees. Further research should explore whether there is a difference in decision‐making preferences between people with low health literacy and adequate health literacy, and focus more on what exactly constitutes and leads to “uninformed” decision making.

### 4.1 Strengths & limitations

There are several limitations to this study. First, the majority of the individuals eligible for CRC screening in this study had not been invited for CRC screening yet. The interview questions about decision‐making processes were therefore mostly hypothetical. However, this also ensured that participants’ statements about their decision making were not influenced by screening outcomes. Four participants had already participated in CRC screening. We did not exclude those individuals from participation in our study, as we were interested in the process of decision making and not necessarily in personal attitudes or experiences regarding CRC screening participation. To ensure that we were uncovering perceptions about the decision‐making process, MF discussed the information materials with participants at the beginning of the focus group interviews to clarify any misunderstandings. Accordingly, we tried to mimic the actual invitation procedure by giving participants the invitation materials from the National Institute of Public Health and the Environment (RIVM). It should be noted that our study population is likely to be biased towards people with a positive attitude towards CRC screening and may therefore report fewer barriers to CRC screening. With regard to the focus groups with experts, it is important to mention that most professionals are experts in the field of shared decision making (SDM), in which the health‐care provider plays an important role. Even though the experts were well aware of the differences between SDM and IDM, this sometimes led to confusion as there is no direct involvement of a health‐care provider in the national CRC screening programme.

## 5 CONCLUSIONS

This study provides insight into the decision‐making stages and its corresponding health literacy skills that are essential for IDM in CRC screening. Our findings show important differences in perceptions between individuals eligible for CRC screening and experts on these decision‐making stages and skills. The conceptual framework can be used to inform the development of context‐based measurement of health literacy and interventions to support IDM in cancer screening.

## CONFLICT OF INTERESTS

We declare that we have no conflict of interests.
